# Positional Patterns Among the Auriculotemporal Nerve, Superficial Temporal Artery, and Superficial Temporal Vein for use in Decompression Treatments for Migraine

**DOI:** 10.1038/s41598-018-34765-1

**Published:** 2018-11-08

**Authors:** Hyung-Jin Lee, You-Jin Choi, Kang-Woo Lee, Hee-Jin Kim

**Affiliations:** 1 Division in Anatomy and Developmental Biology, Department of Oral Biology, Human Identification Research Institute, BK21 PLUS Project, Yonsei University College of Dentistry, Seoul, South Korea; 2 Department of Anatomy, Yonsei University College of Medicine, Seoul, South Korea; 3 Department of Materials Science & Engineering, College of Engineering, Yonsei University, Seoul, South Korea

## Abstract

This study aimed to clarify intersection patterns and points among the superficial temporal artery (STA), superficial temporal vein (STV), and auriculotemporal nerve (ATN) based on surface anatomical landmarks to provide useful anatomical information for surgical decompression treatments of migraine headaches in Asians. Thirty-eight hemifaces were dissected. The positional patterns among the ATN, STA, and STV were divided into three morphological types. In type I, the ATN ran toward the temporal region and superficially intersected the STA and STV (n = 32, 84.2%). In type II, the ATN ran toward the temporal region and deeply intersected the STA and STV (n = 4, 10.5%). In type III, the ATN ran toward the temporal region and deeply intersected the STV alone (n = 2, 5.3%). The intersection points of types II and III were 10.3 ± 5.6 mm (mean ± SD) and 10.4 ± 6.1 mm anterior and 42.1 ± 21.6 mm and 41.4 ± 18.7 mm superior to the tragus, respectively. The ATN superficially intersected the STA and STV in all the Korean cadaver, while the ATN deeply intersected the STA and STV in 15% of the Thai cadavers. The pattern of the ATN deeply intersecting the STA and STV was less common in present Asian populations than in previously-reported Caucasian populations, implying that migraine headaches (resulting from the STA and STV compressing the ATN) are less common in Asians.

## Introduction

Until recently it was thought that migraine headaches were centrally mediated within the brain^1^. However, since 2000 the pathway of the peripheral nerves has been increasingly accepted as the cause of migraine headaches in the head and neck regions^2^. Migraines in the head and neck may occur along the course of the branches of the trigeminal nerve and the cervical spinal nerve(s), whose branches distribute to the posterior part of the neck. Such migraines resulting from this novel concept are reported to be treatable using various surgical and nonsurgical methods^3–5^.

The supratrochlear and supraorbital nerves are peripheral branches of the trigeminal nerve that may be compressed where they pass the corrugator supercilii muscle in the forehead area^6–11^. That compression point is considered to be a peripheral trigger point for migraine headaches, and previous studies found that complete or partial corrugator resection and surgical decompression of the nerve provided significant migraine relief in 75–80% of patients^4,5,12,13^. Various clinical trials and accumulating evidence are strengthening support for the theory of peripheral trigger points for migraine in the clinical field^14^.

However, a patient has been reported who did not respond to this treatment despite his symptom being observed in the temporal region^15^. In the temporal region, the zygomaticotemporal nerve is generally known to be the main target nerve for treatments of temporal migraine headache^16,17^. However, it is reported that the patient had a migraine along the course of the auriculotemporal nerve (ATN) in the temple region^15^. Also, a previous research study had determined that the most-likely anatomical factor was the positional relationship between a vessel and the nerve^15,18^. Some clinicians including neurologist have observed that a vessel running superficial to the nerve can produce a migraine headache by compressing the underlying nerve. Various anatomical and clinical studies have focused on the relationship between the artery and the nerve^15,16,18^, resulting in the superficial temporal artery (STA) reportedly running superficial to the ATN in 80% of Caucasians in the temporal area. The patient who exhibited the above-mentioned anatomical structure required surgical decompression to relieve the migraine headache^16^. However, few anatomical studies have focused on the positional pattern among the ATN and the superficial temporal vessels or its crossing points and patterns at the temporal region in Asians, with the aim of determining how to relieve migraine headaches via surgical decompression.

The aim of this study was to clarify the intersection patterns and points among the STA, superficial temporal vein (STV), and ATN based on surface anatomical landmarks in Asians. Furthermore, the intersection patterns and points of the neurovascular structure in the temporal area were compared with Caucasian anatomical data in order to provide effective anatomical information for surgical decompression in Asians.

## Materials and Methods

**Figure 1:**
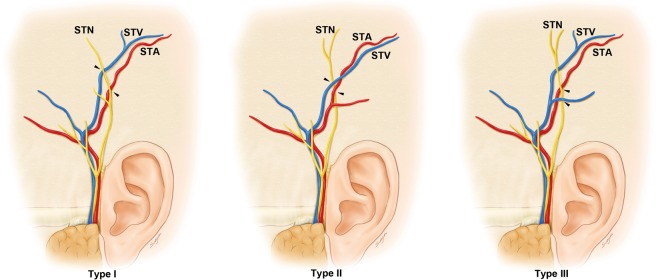
Illustrations showing the intersection points (arrowheads) of the three patterns of the auriculotemporal nerve (ATN), superficial temporal artery (STA), and superficial temporal vein (STV) based on morphology. Those in the photographs are all male. These figures were drawn by Su Hyun Chae.

**Figure 2:**
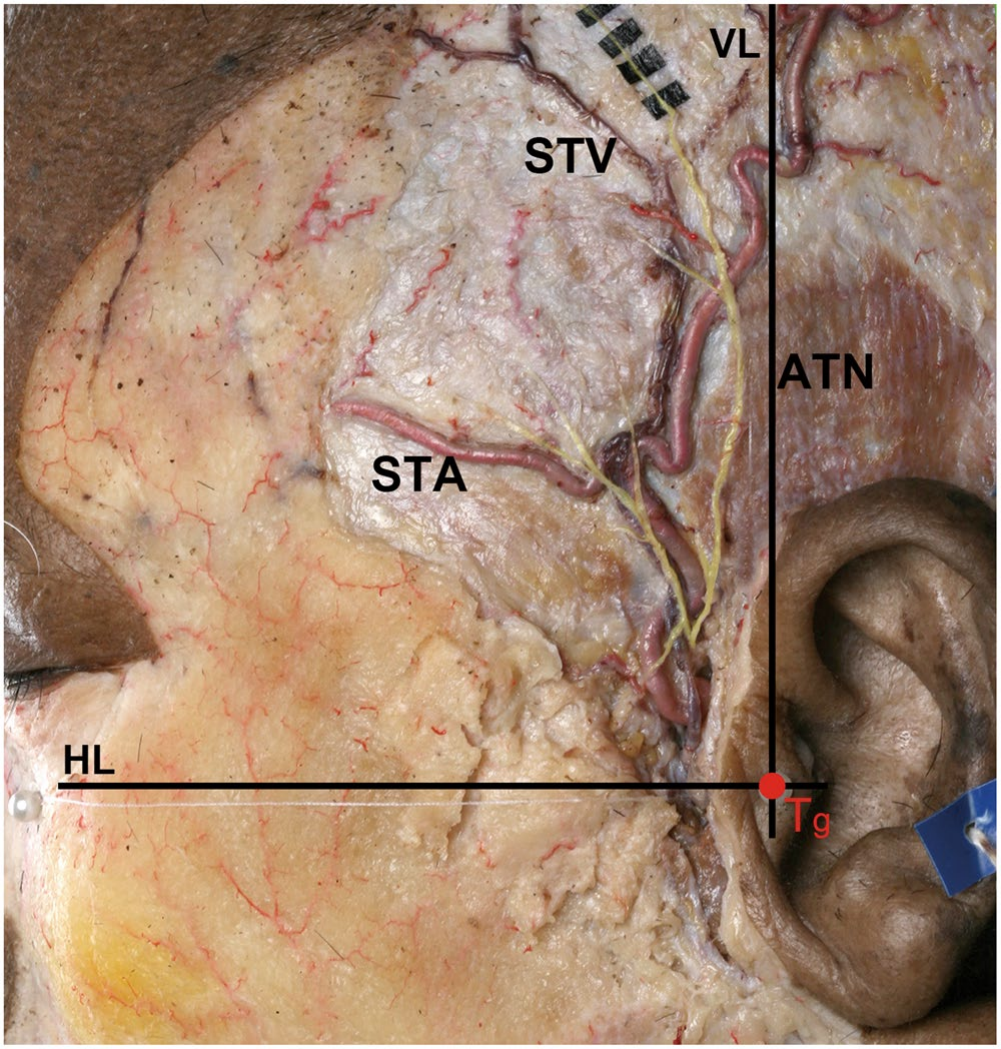
The reference planes used for measuring the intersection points of the ATN, STA, and STV. The line passing transversely through the tragus (Tg) is labeled HL. The line perpendicular to reference plane H and passing through Tg is labeled VL.

All experimental procedures were performed in accordance with the Declaration of Helsinki of the World Medial association (WMA). After the approval of the Surgical Anatomy Education Centre, Yonsei University College of Medicine and the Chula Soft Cadaver Surgical Training Center, Faculty of Medicine, Chulalongkorn University, all thirty-eight hemifaces were legally donated to these institutes, and subjected to the dissection of the temporal region (28 embalmed [15 males, 13 females]; mean age, 78.3 years; 15 specimens were from the College of Dentistry, Yonsei University, Seoul, Korea; 13 specimens were from the Faculty of Medicine, Chulalongkorn University, Bangkok, Thailand). The consent to donation of the bodies for research purposes was given by the subjects^11^. The skin was delicately removed from the temporal region. The subcutaneous layer, superficial musculoaponeurotic system, and auricular muscle were also dissected to reveal the ATN, STA, and STV. First, the positional relationships among the ATN, STA, and STV were observed in the temple region of the dissected specimens. These relationships were divided into the three types based on morphology (Fig. 1). Second, the intersection point, where the ATN deeply intersects the STA and STV, was measured based on the following two reference planes (Fig. 2).

Plane H: the horizontal plane through the Tg and the lowermost point of the inferior orbital rim (modified Frankfort line).

Plane V: the vertical plane perpendicular to reference plane H that passes through the Tg.

The diameters of the ATN, STA, and STV were measured at their intersection points. Digital calipers (Catalog No. 500-196-20, Mitutoyo, Kanagawa, Japan) were used to measure both distances and diameters. Microsoft Excel software (version 2016, Microsoft, Redmond, WA, USA) was used for data analysis.

## Results

### Intersection patterns among the ATN and superficial temporal vessels

The positional relationships among the ATN, STA, and STV were divided into the following three types based on morphology (Fig. 1).

Type I: The ATN runs toward the temporal region and superficially intersects the STA and STV.

Type II: The ATN runs toward the temporal region and deeply intersects the STA and STV.

Type III: The ATN runs toward the temporal region and deeply intersects the STV alone.

**Figure 3:**
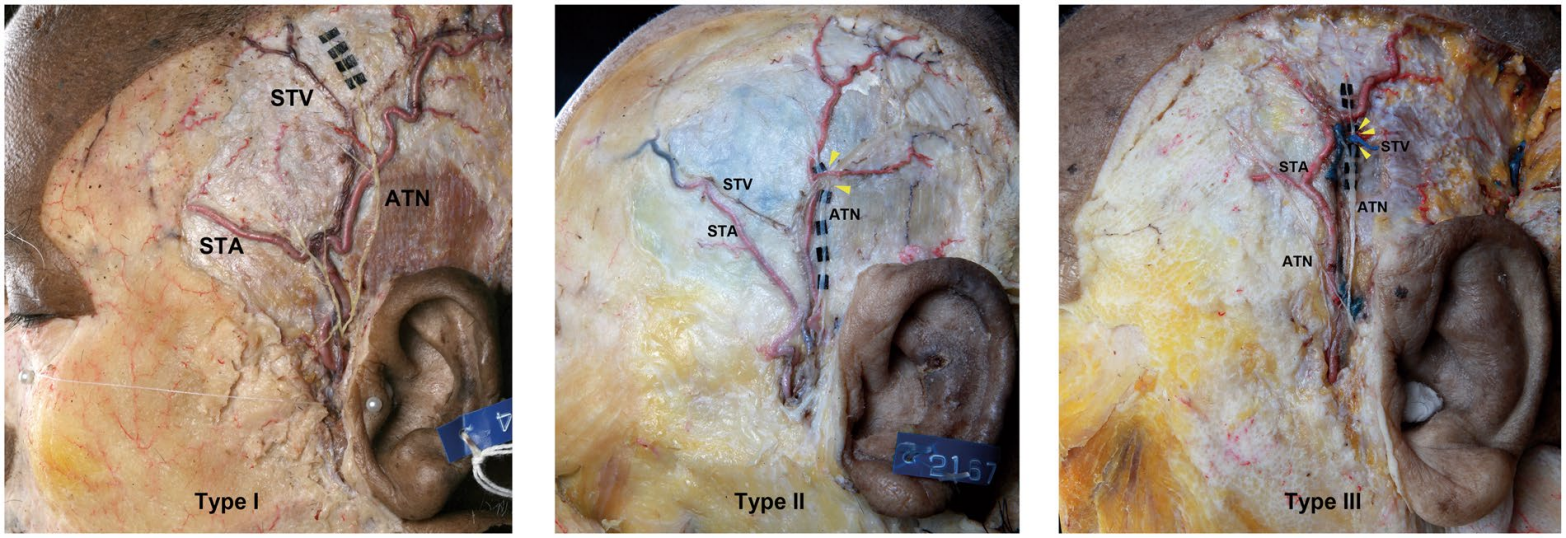
Photographs showing examples of the positional relationships among the ATN, STA, and STV. A, Type I, where the ATN superficially intersects the STA and STV. B, Type II, where the ATN deeply intersects the STA and STV. C, Type III, where the ATN deeply intersects the STV alone. Yellow arrowheads show the deep intersection point of the ATN in relation to the STA and STV.

The ATN, STA, and STV were dissected and identified in all specimens. The intersection pattern among the ATN and superficial temporal vessels was classified into three types (Fig. 3): type I, in which the ATN ran toward the temporal region and superficially intersected the STA and STV (*n* = 32, 84.2%); type II, in which the ATN ran toward the temporal region and deeply intersected the STA and STV (*n* = 4, 10.5%); and type III, in which the ATN ran toward the temporal region and deeply intersected the STV alone (*n* = 2, 5.3%) (Fig. 3). The ATN superficially intersected the STA and STV in all of the Korean cadavers, while it deeply intersected the STA and STV in 15% of the Thai cadavers.

### Compression point of the ATN in the temporal area

**Figure 4:**
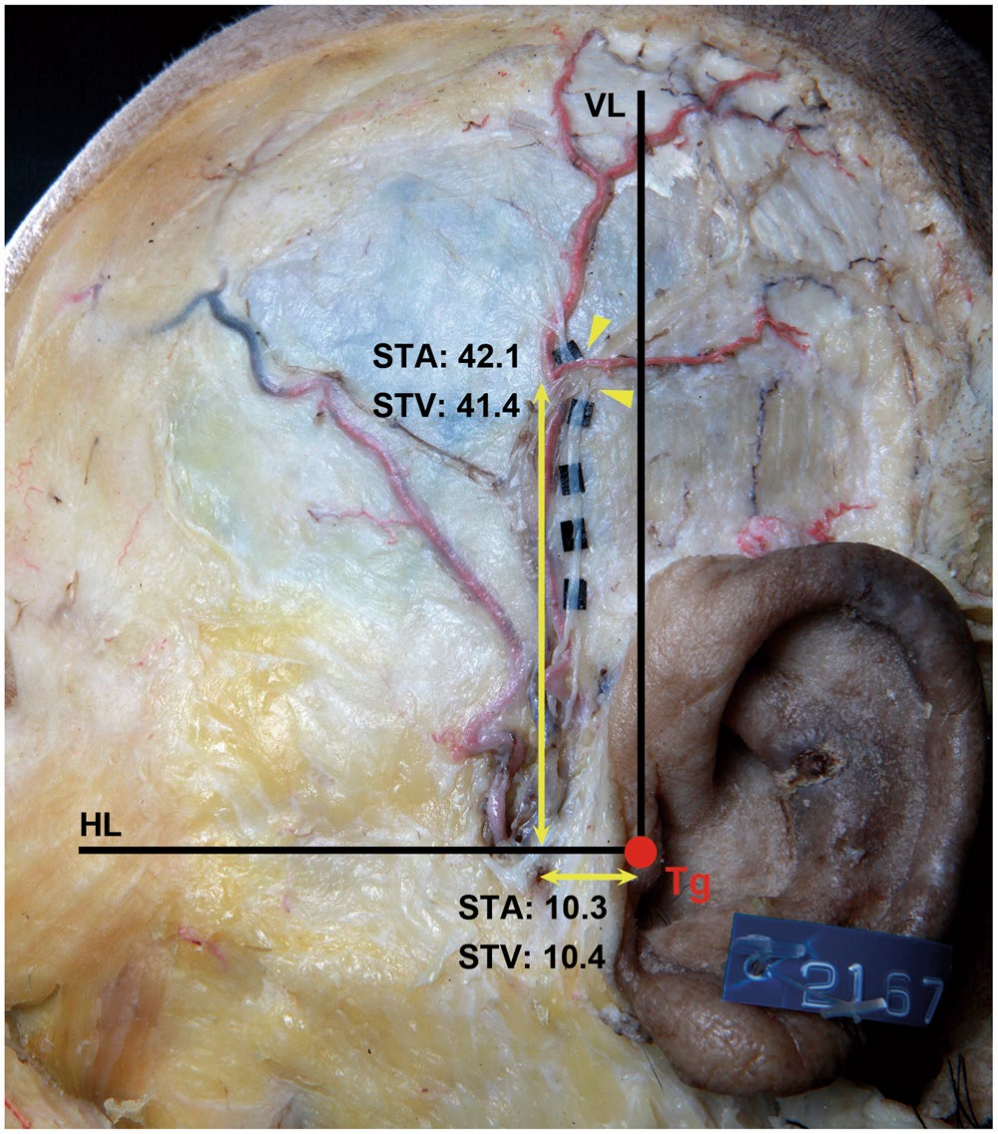
Photograph showing the measured intersection points among the ATN, STA, and STV based on reference lines. The ATN intersected with the STA and STV at 10.3 mm and 10.4 mm anterior to reference plane V, and at 42.1 mm and 41.4 mm superior to reference plane H, respectively. Dimensions are in millimeters.

The points at which the ATN intersected with the STA and STV were measured based on reference planes H and V (see Methods) in types II and III (Fig. 4). Also, the diameters of the ATN, STA, and STV were measured at their intersection points. In type II specimens, the point at which the ATN intersected the STA was 10.3 ± 5.6 mm (mean ± SD) anterior to plane V and 42.1 ± 21.6 mm superior to plane H. In type III specimens, the ATN intersected the STV at 10.4 ± 6.1 mm anterior to plane V and 41.4 ± 18.7 mm superior to plane H (Table 1). The diameters of the ATN, STA, and STV where the ATN intersected the STA and STV in the temporal region were 0.8 ± 0.2 mm, 1.6 ± 0.5 mm, and 2.0 ± 0.2 mm, respectively (Table 2).

**Table 1:** Intersection points among the ATN, STA, and STV in the temporal region.

	Intersection of ATN-STA		Intersection of ATN-STV	
**unit: mm**				
	X	Y	X	Y
Average	10.3	42.1	10.4	41.4
SD	5.6	21.6	6.1	18.7

ATN, auriculotemporal nerve; STA, superficial temporal artery; STV, superficial temporal vein.

**Table 2:** Diameter of the ATN, STA, and STV at intersection point in the temporal region.

	Measured anatomical structures		
**unit: mm**			
	ATN	STA	STV
Average	0.8	1.6	2.0
SD	0.2	0.5	0.2

ATN, auriculotemporal nerve; STA, superficial temporal artery; STV, superficial temporal vein.

## Discussion

The ATN and the zygomaticotemporal nerve are branches of the trigeminal nerve, and they distribute into the mandibular and maxillary nerves, respectively. These two nerves are responsible for sensations in the entire temporal area^19^. The zygomaticotemporal nerve is considered the main trigger site for migraine headaches in the temporal area at an anatomical level^16,17^. Furthermore, the ATN has recently been considered a possible trigger site for migraine headaches, prompting numerous studies investigating this by focusing on the course of the ATN and its relationship with the STA in Caucasians^15,16,18^. Though surgical decompression of the ATN can reportedly significantly improve migraine headaches (up to 90% of success rate) in Caucasians populations, there are few anatomical data on Asians regarding the relationship among the ATN, STA, and the STV in the temporal area. This study, therefore, investigated the relationships among the ATN, STA, and STV, and examined the intersection patterns and points for the purpose of predicting potential trigger points for migraine headaches and providing anatomical information for applications of surgical decompression on Asian populations.

The ATN ascends posterior to the superficial temporal vessels and supplies the posterior part of the temple. The above-mentioned neurovascular structure has a direct relationship with each other which results in migraine headaches by irritating, entrapping, or compressing at multiple points along their anatomical course. While the ATN was a distinct nerve with a diameter of 0.8 mm at the intersection point between the ATN and superficial temporal vessels, detection rate of the ATN have varied considerably, ranging from 34% to 80% among previous studies^15,16,18^. In the present study, we observed that the ATN superficially intersected the STA and STV in 81.3% of the specimens while the ATN deeply intersected the STA and STV at a single point in the temporal area in 15.8% of the specimens. These results contrast with Chim *et al*.^15^ who reported that the ATN deeply intersected the STA in 80% of their Caucasian specimens and crossed the STA at a single point in the temporal area. In addition, the results contrast with Janis *et al*.’s^18^ report that the ATN deeply intersected the STA in 34% of their Caucasian specimens. Based on these anatomical information on Caucasian and Asian populations, we propose that the ATN could be compressed, irritated or entrapped by the superficial temporal vessels when it has a close relationship with STA and STV.

Until now, previous anatomical studies showed that the ATN has a direct relationship only with the STA in temporal area. However, in the present study, the ATN deeply intersected the STV in 5.3% (2 of 38 cases). In some case, bulging veins are typically observed at the forehead and temple area. Large bulging veins are often associated with age or genetics. When the vein pressure increases by frequent sneezing, exercise, or severe vomiting, the veins expand, which can cause the vein to bulge^20^. Therefore, since the ATN interacted with the STV could also be regarded as a potential compressing anatomical structure that results in migraine headaches in the temporal region for Asian populations.

Even among Asian populations, we specifically observed the different anatomical variations between Korean and Thai cadavers. The ATN superficially intersected the STA and the STV in all the Korean cadaver while the ATN deeply intersected the STA and STV in 15% of the Thai cadavers. Various previous anatomic studies described ethnic differences with regard to presence of the supraorbital notch or foramen, the supraorbital nerve exit from the orbit, and morphology of corrugator supercilii muscle^7–9,21–24^ but, not with regard to the intersection pattern among the ATN, STA, and STV in the temporal area. Hence, neurologists and clinicians must be aware of differences in the morphological intersection patterns of the ATN and the superficial temporal vessels between Asians and Caucasians—even within Asian populations—in order to optimize the results obtained when performing surgical decompression of the ATN in the temporal area.

In general, the most acute pain site depended on the patient’s guidance or on the course of the nerve prior to surgical decompression. Guyuron *et al*. recently demonstrated that an ultrasound Doppler signaling technique could lead to successful identification of the ATN following the patient’s pointing to the area of most acute pain site. This doppler signaling technique allowed the clinician to detect exact compression/irritation site in every patient and to make small incision to release the migraine headache^16^. However, not all the patients may always feel pain at the time of surgery. Also, there is no anatomical information regarding the intersection point among the ATN, STA, and STV or compression point in Asian populations that results in migraine headaches in the temporal area. In the present study, the position of the potential compression point was determined by measuring where the ATN intersected the STA from the tragus (Tg). In type II specimens, the ATN intersected the STA at 10.3 ± 5.6 mm anterior and 42.1 ± 21.6 mm superior to the Tg. In type III specimens, the ATN intersected the STV at 10.4 ± 6.1 mm anterior and 41.4 ± 18.7 mm superior to the Tg (Fig. 4). Chim *et al*. set the most-anterosuperior point of the external meatus of the ear as the reference point when measuring the intersection point of the ATN and STA and found that the ATN intersected the STA at 19.2 ± 10.0 mm anterior and 39.5 ± 16.6 mm superior to the reference point^15^. However, the reference point slightly differs between the present study and that of Chim *et al*. The Tg and the most-anterosuperior point of the external meatus could be considered the same reference point. So, while the intersection patterns varied between Asians and Caucasians, the intersection point—corresponding to the potential compression point—appears to be very similar in the two races.

Chim *et al*. found that the potential compression point of the ATN was indicated in the superior preauricular region by a fascial band^15^. However, this band was not observed in the present study. Hence, we believe that migraine headaches in Asians are more likely to occur where the ATN intersects the STA rather than where the ATN is compressed by the fascial band.

Knowledge of the compression point at each region is important to treat migraine headaches. The mechanisms of peripherally mediated migraine headache vary among the regions. The four main peripheral trigger sites are frontal, temporal, septal/turbinates and occipital region^15^. The fascial band of the supraorbital notch and corrugator supercilii muscle and the orbital septum might be potential anatomical structures that cause migraine headache in forehead region^11^. These structures are known to cause the migraine headache by entrapping nerves when the supraorbital and supratrochlear emerge from the orbit and pierce the muscle^6,7,9^. The temporalis and the STA from the temporal region and semispinalis capitis, trapezius, and occipital artery from the occipital region are also the potential anatomical structure that cause the migraine headaches^15,17,18,25–28^. All above-mentioned structures are located in the vicinity of the nerve that causes the migraine headaches. The compression points of the frontal, temporal, septal/turbinates and occipital region in Caucasians are well demonstrated according to the numerous anatomical and clinical studies^3–5,7–12,14,15,17,18,26,28^. However, there are no anatomical and clinical studies focusing on the compression point of the temporal region in Asian populations. Therefore, the anatomical data obtained herein would be significantly helpful in predicting the compression point in the temporal area for those who experience a migraine headache along the course of the ATN.

The Tg is remarkable structure of the ear. Given that the tragus is obvious surface landmark among the several superficial structures of the ear, the position of the intersection points between ATN and superficial temporal vessels (i.e., STA and STV) in relation to Tg may allow a clinician to estimate the trigger point for migraine headaches prior to surgical decompression of the ATN. In the present study, the ATN intersected the superficial temporal vessels at an average of 10 mm anterior and 40 mm superior to the Tg. Also, on average, Chim *et al*. reported that the ATN intersected the STA at 19 mm anterior and 39 mm superior to the external meatus of the ear^15^. Since the intersection points between the ATN and superficial temporal vessels are similar in Caucasian and Asian populations, the Tg could be regarded as a significant surface landmark to identify the compression/irritation point in the temporal area for both Caucasian and Asian ethnic groups.

## Conclusion

In conclusion, we have provided new and useful anatomical data through dissections of the ATN, STA, and STV in the temporal regions of Asian populations which contrasts with previous studies predominantly focusing on Caucasian Americans. The pattern in which the ATN deeply intersects the STA and STV was less common in our Asian populations (only 15.8% of the specimens) than in previous Caucasian populations, suggesting that migraine headaches resulting from compression of the ATN by the STA and STV would be less common in Asians. Moreover, the present findings could be helpful reference data for anatomically understanding migraine headaches in the temporal area. The anatomical data regarding the positional relationships among the ATN, STA, and STV obtained in the present study might be useful for predicting and alleviating potential factors that may lead to migraine headaches and for performing surgical decompressions in the temporal region, especially in Asian populations.

## Data Availability

The datasets generated during and/or analyzed during the current study are available from the corresponding author on reasonable request.
